# Supplementary material for: Drug repurposing for aging research using model organisms
Source: Aging Cell. 2017 Jun 16;16(5):1006–15. doi: 10.1111/acel.12626 (PMC5595691; doi:10.1111/acel.12626)
Supplement: Supplementary file 7 — Data S1 Zip‐Archive of all report cards. [file ACEL-16-1006-s007.zip › RC_MLT.pdf]

MLT

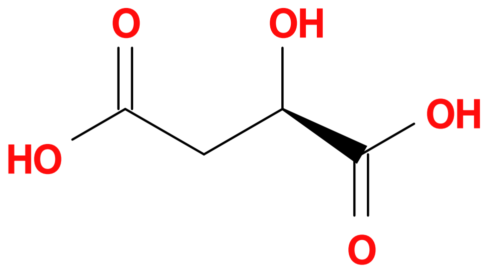

Database identifiers

ChEMBLCompound	CHEMBL225986
CHEBI	30796
eMolecules	475035

Ranking

	Rank	Score
Drosophila	315/697	0.5
C. elegans	297/591	0.194

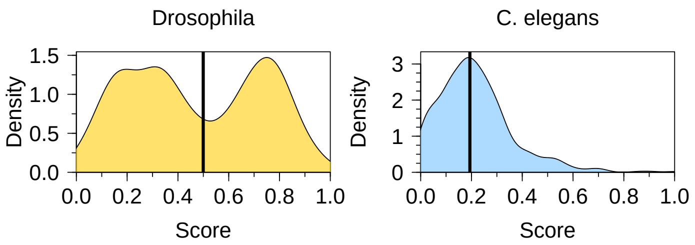

	Ageing implication	Domain conservation	Binding site conservation	Binding affinity	Bioavailability	Lipinski	Promiscuity	Purchasability	Drug approval	**Total**
Drosophila	1.0	0.904	1.0	0.493	(0.9)	0.0	-0.001	0.1	0.0	0.5
C. elegans	1.0	0.914	1.0	0.493	0.211	0.0	-0.001	0.1	0.0	0.194

Names

- **(R)-malic acid**
- (R)-2-hydroxybutanedioic acid
- 2-hydroxy-succinic acid

Roles

ChEBI entry 30796 has no roles

Status

Approved drug (according to ChEMBL)	No
Number of Rule of 5 violations	0
Binding affinity to original target in log units (RF-Score prediction)	4.97
Burns *C. elegans* bioavailability prediction	-5.65

Compound Target Characteristics

NAD-dependent malic enzyme, mitochondrial

Best gene implication in ageing for this target family came from gene Q9NIW0 annotated in UniProt release 2014_02. Annotation GO 8340 (determination of adult lifespan) was Inferred from Mutant Phenotype

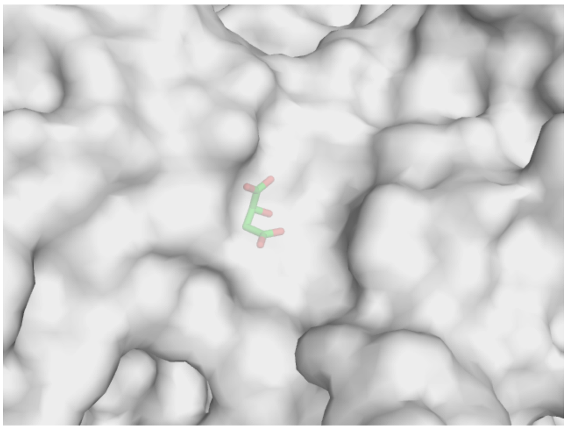

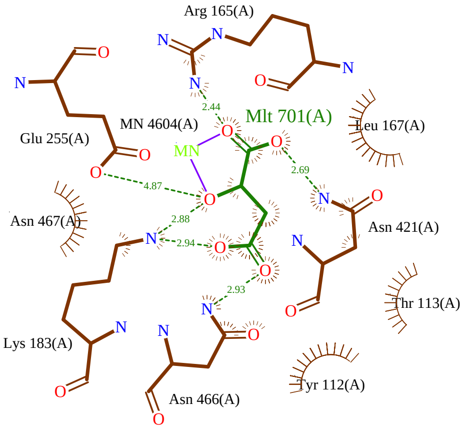

protein	amino acids contacts (binding site)
PDB:1pj2:chainA:P23368	Y T R L K E D D N N N
PDB:1pj4:chainA:P23368	Y T R L K E D D N N N
tr:B4DZ70:B4DZ70_HUMAN	Y T R L K E D D N N N
sp:P48163:MAOX_HUMAN	Y T R L K E D D N N N
tr:E9PMB9:E9PMB9_HUMAN	Y T R L K E D D N N N
sp:P23368:MAOM_HUMAN	Y T R L K E D D N N N
sp:Q16798:MAON_HUMAN	Y T R L K E D D N N N
tr:F1LQQ1:F1LQQ1_RAT	Y T R L K E D D N N N
tr:D3ZJH9:D3ZJH9_RAT	Y T R L K E D D N N N
tr:F1M5N4:F1M5N4_RAT	Y T R L K E D D N N N
sp:P06801:MAOX_MOUSE	Y T R L K E D D N N N
sp:Q99KE1:MAOM_MOUSE	Y T R L K E D D N N N
sp:Q8BMF3:MAON_MOUSE	Y T R L K E D D N N N
tr:H7BWR6:H7BWR6_DROME	Y T R L K E D D N N N
tr:Q9NIW0:Q9NIW0_DROME	Y T R L K E D D N N N
tr:H7BWR4:H7BWR4_DROME	Y T R L K E D D N N N
tr:H7BWU6:H7BWU6_DROME	Y T R L K E D D N N N
tr:H7BWS3:H7BWS3_DROME	Y T R L K E D D N N N
tr:H7BWS2:H7BWS2_DROME	Y T R L K E D D N N N
tr:Q2QBM1:Q2QBM1_DROME	Y T R L K E D D N N N
tr:H7BWS7:H7BWS7_DROME	Y T R L K E D D N N N
tr:H7BWS6:H7BWS6_DROME	Y T R L K E D D N N N
tr:H7BWU8:H7BWU8_DROME	Y T R L K E D D N N N
tr:Q9U1J1:Q9U1J1_DROME	Y T R L K E D D N N N
tr:Q9NIW1:Q9NIW1_DROME	Y T R L K E D D N N N
tr:Q9VG32:Q9VG32_DROME	Y T R L K E D D N N N
tr:Q9NIW2:Q9NIW2_DROME	Y T R L K E D D N N N
tr:Q9VG31:Q9VG31_DROME	Y T R L K E D D N N N
tr:Q9U296:Q9U296_CAEEL	Y T R L K E D D N N N
sp:P36013:MAOM_YEAST	Y T G L K E D D N N N

	whole protein		domain-based		contact-based	
protein	ident	simil	ident	simil	ident	simil
PDB:1pj2:chainA:P23368	1.0	1.0	1.0	1.0	1.0	1.0
PDB:1pj4:chainA:P23368	1.0	1.0	1.0	1.0	1.0	1.0
tr:B4DZ70:B4DZ70_HUMAN	0.48	0.73	0.51	0.77	1.0	1.0
sp:P48163:MAOX_HUMAN	0.53	0.83	0.56	0.86	1.0	1.0
tr:E9PMB9:E9PMB9_HUMAN	0.51	0.79	0.55	0.84	1.0	1.0
sp:P23368:MAOM_HUMAN	1.0	1.0	1.0	1.0	1.0	1.0
sp:Q16798:MAON_HUMAN	0.52	0.82	0.56	0.86	1.0	1.0
tr:F1LQQ1:F1LQQ1_RAT	0.52	0.81	0.55	0.85	1.0	1.0
tr:D3ZJH9:D3ZJH9_RAT	0.89	0.97	0.9	0.97	1.0	1.0
tr:F1M5N4:F1M5N4_RAT	0.5	0.8	0.54	0.85	1.0	1.0
sp:P06801:MAOX_MOUSE	0.53	0.83	0.56	0.86	1.0	1.0
sp:Q99KE1:MAOM_MOUSE	0.88	0.96	0.9	0.97	1.0	1.0
sp:Q8BMF3:MAON_MOUSE	0.53	0.82	0.57	0.87	1.0	1.0
tr:H7BWR6:H7BWR6_DROME	0.47	0.77	0.5	0.82	1.0	1.0
tr:Q9NIW0:Q9NIW0_DROME	0.46	0.76	0.5	0.82	1.0	1.0
tr:H7BWR4:H7BWR4_DROME	0.46	0.76	0.5	0.82	1.0	1.0
tr:H7BWU6:H7BWU6_DROME	0.45	0.76	0.5	0.82	1.0	1.0
tr:H7BWS3:H7BWS3_DROME	0.46	0.76	0.5	0.82	1.0	1.0
tr:H7BWS2:H7BWS2_DROME	0.46	0.76	0.5	0.82	1.0	1.0
tr:Q2QBM1:Q2QBM1_DROME	0.46	0.76	0.5	0.82	1.0	1.0
tr:H7BWS7:H7BWS7_DROME	0.45	0.76	0.5	0.82	1.0	1.0
tr:H7BWS6:H7BWS6_DROME	0.45	0.76	0.5	0.82	1.0	1.0
tr:H7BWU8:H7BWU8_DROME	0.46	0.76	0.5	0.82	1.0	1.0
tr:Q9U1J1:Q9U1J1_DROME	0.44	0.74	0.48	0.8	1.0	1.0
tr:Q9NIW1:Q9NIW1_DROME	0.36	0.61	0.5	0.82	1.0	1.0
tr:Q9VG32:Q9VG32_DROME	0.36	0.61	0.5	0.82	1.0	1.0
tr:Q9NIW2:Q9NIW2_DROME	0.36	0.61	0.5	0.82	1.0	1.0
tr:Q9VG31:Q9VG31_DROME	0.36	0.61	0.5	0.82	1.0	1.0
tr:Q9U296:Q9U296_CAEEL	0.47	0.78	0.51	0.84	1.0	1.0
sp:P36013:MAOM_YEAST	0.3	0.65	0.34	0.74	0.91	0.88

UniProt:H7BWR6 annotation

Cofactor: Mg(2+) Evidence=(PIRSR:PIRSR000106-3); Name=Mn(2+); Xref=ChEBI:CHEBI:29035; Evidence=(PIRSR:PIRSR000106-3); Note=Divalent metal cations. Prefers magnesium or manganese. (PIRSR:PIRSR000106-3);

(Information from UniProt)

Men (FBgn0002719) associated phenotypes

fast, intermediate, long lived, poor, sleep defective

(Information from FlyBase)

Men (UniProt:Q9NIW0) annotation

Cofactor: Mg(2+) Evidence=(PIRSR:PIRSR000106-3); Name=Mn(2+); Xref=ChEBI:CHEBI:29035; Evidence=(PIRSR:PIRSR000106-3); Note=Divalent metal cations. Prefers magnesium or manganese. (PIRSR:PIRSR000106-3);

(Information from UniProt)

UniProt:H7BWR4 annotation

Cofactor: Mg(2+) Evidence=(PIRSR:PIRSR000106-3); Name=Mn(2+); Xref=ChEBI:CHEBI:29035; Evidence=(PIRSR:PIRSR000106-3); Note=Divalent metal cations. Prefers magnesium or manganese. (PIRSR:PIRSR000106-3);

(Information from UniProt)

UniProt:H7BWU6 annotation

Cofactor: Mg(2+)Name=Mn(2+); Xref=ChEBI:CHEBI:29035; Note=Divalent metal cations. Prefers magnesium or manganese.;

(Information from UniProt)

UniProt:H7BWS3 annotation
Cofactor: Mg(2+) Evidence=(PIRSR:PIRSR000106-3); Name=Mn(2+); Xref=ChEBI:CHEBI:29035; Evidence=(PIRSR:PIRSR000106-3); Note=Divalent metal cations. Prefers magnesium or manganese. (PIRSR:PIRSR000106-3);

(Information from UniProt)

UniProt:H7BWS2 annotation
Cofactor: Mg(2+) Evidence=(PIRSR:PIRSR000106-3); Name=Mn(2+); Xref=ChEBI:CHEBI:29035; Evidence=(PIRSR:PIRSR000106-3); Note=Divalent metal cations. Prefers magnesium or manganese. (PIRSR:PIRSR000106-3);

(Information from UniProt)

Men (UniProt:Q2QBM1) annotation
Cofactor: Mg(2+)Name=Mn(2+); Xref=ChEBI:CHEBI:29035; Note=Divalent metal cations. Prefers magnesium or manganese.;

(Information from UniProt)

UniProt:H7BWS7 annotation
Cofactor: Mg(2+) Evidence=(PIRSR:PIRSR000106-3); Name=Mn(2+); Xref=ChEBI:CHEBI:29035; Evidence=(PIRSR:PIRSR000106-3); Note=Divalent metal cations. Prefers magnesium or manganese. (PIRSR:PIRSR000106-3);

(Information from UniProt)

UniProt:H7BWS6 annotation
Cofactor: Mg(2+) Evidence=(PIRSR:PIRSR000106-3); Name=Mn(2+); Xref=ChEBI:CHEBI:29035; Evidence=(PIRSR:PIRSR000106-3); Note=Divalent metal cations. Prefers magnesium or manganese. (PIRSR:PIRSR000106-3);

(Information from UniProt)

UniProt:H7BWU8 annotation
Cofactor: Mg(2+) Evidence=(PIRSR:PIRSR000106-3); Name=Mn(2+); Xref=ChEBI:CHEBI:29035; Evidence=(PIRSR:PIRSR000106-3); Note=Divalent metal cations. Prefers magnesium or manganese. (PIRSR:PIRSR000106-3);

(Information from UniProt)

Men (UniProt:Q9U1J1) annotation
Cofactor: Mg(2+) Evidence=(PIRSR:PIRSR000106-3); Name=Mn(2+); Xref=ChEBI:CHEBI:29035; Evidence=(PIRSR:PIRSR000106-3); Note=Divalent metal cations. Prefers magnesium or manganese. (PIRSR:PIRSR000106-3);

(Information from UniProt)

men-1 (UniProt:Q9U296) annotation
Cofactor: Mg(2+) Evidence=(PIRSR:PIRSR000106-3); Name=Mn(2+); Xref=ChEBI:CHEBI:29035; Evidence=(PIRSR:PIRSR000106-3); Note=Divalent metal cations. Prefers magnesium or manganese. (PIRSR:PIRSR000106-3);

(Information from UniProt)

Tyrosine-protein phosphatase non-receptor type 11

Best gene implication in ageing for this target family came from gene G5EC24 via mapping the annotation from WormBase WBGene00004214 annotated in WormBase 2014-03-11. Annotation

GO 8340 (determination of adult lifespan) was Inferred from Mutant Phenotype

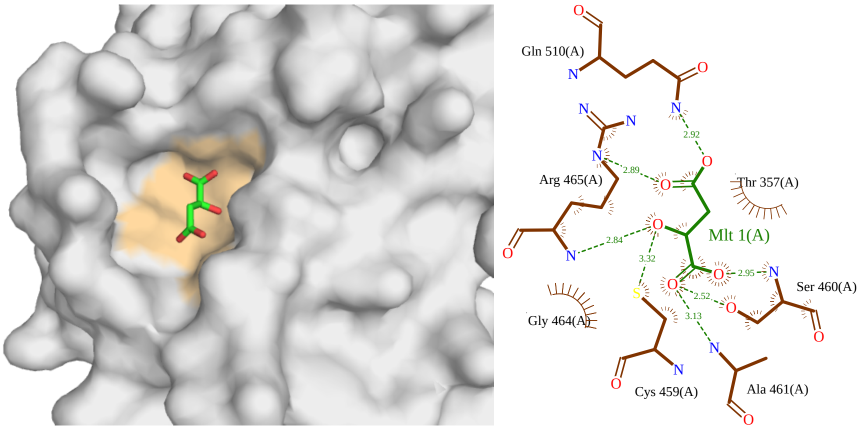

protein	amino acids contacts (binding site)
PDB:3b7o:chainA:Q06124	T C S A G R Q
sp:Q06124:PTN11_HUMAN	T C S A G R Q
sp:P41499:PTN11_RAT	T C S A G R Q
sp:P35235:PTN11_MOUSE	T C S A G R Q
sp:P29349:CSW_DROME	T C S A G R Q
tr:G5EC24:G5EC24_CAEEL	T C S A G R Q

	whole protein		domain-based		contact-based	
protein	ident	simil	ident	simil	ident	simil
PDB:3b7o:chainA:Q06124	1.0	1.0	1.0	1.0	1.0	1.0
sp:Q06124:PTN11_HUMAN	1.0	1.0	1.0	1.0	1.0	1.0
sp:P41499:PTN11_RAT	0.99	1.0	0.99	1.0	1.0	1.0
sp:P35235:PTN11_MOUSE	0.99	1.0	1.0	1.0	1.0	1.0
sp:P29349:CSW_DROME	0.41	0.57	0.39	0.57	1.0	1.0
tr:G5EC24:G5EC24_CAEEL	0.32	0.64	0.37	0.66	1.0	1.0

csw (FBgn0000382) associated phenotypes

dominant, germline clone, heat sensitive, increased cell death, lethal - all die before end of pupal stage, maternal effect, rescuable maternal effect, somatic clone, some die during pupal stage

(Information from FlyBase)

csw (UniProt:P29349) annotation

Function: Required in all receptor tyrosine kinase signaling pathways. Functions downstream of the receptor tyrosine kinase torso, acting in concert with D-Raf via tailless. Also functions downstream of Egfr (epidermal growth factor receptor) and btl (fibroblast growth factor receptor). The SH2 domain suggests that csw effects its role by mediating heteromeric protein interactions. Maternally required for normal determination of cell fates at the termini of the embryo. Required for cell fate specification of the ventral ectoderm, in the developing embryonic CNS and for embryonic tracheal cell migration. Functions during imaginal development for proper formation of adult structures such as eyes, aristae, L5 wing vein and the tarsal claw. (PubMed:1638629, PubMed:8948575).
Subcellular location: Cytoplasm.
Tissue specificity: Expressed uniformly throughout all tissues during embryogenesis. (PubMed:1638629).
Developmental stage: Expressed throughout development. (PubMed:1638629).
Miscellaneous: The PTPase domain is interrupted by a PTPase insert which shares no homologies with other PTPase proteins. This PTPase insert is reminiscent of the kinase insert within the kinase catalytic domains of several receptor tyrosine kinases.

(Information from UniProt)

ptp-2 (UniProt:G5EC24) annotation

Subcellular location: Cytoplasm ECO:0000256—PIRNR:PIRNR000929.

(Information from UniProt)
